# Microtubule-based perception of mechanical conflicts controls plant organ morphogenesis

**DOI:** 10.1126/sciadv.abm4974

**Published:** 2022-02-09

**Authors:** Dorothee Stöckle, Blanca Jazmin Reyes-Hernández, Amaya Vilches Barro, Milica Nenadić, Zsofiá Winter, Sophie Marc-Martin, Lotte Bald, Robertas Ursache, Satoshi Fujita, Alexis Maizel, Joop EM Vermeer

**Affiliations:** 1 Department of Plant and Microbial Biology, University of Zurich, Zollikerstrasse 107, 8008 Zurich, Switzerland.; 2 Zurich-Basel Plant Science Center, Zurich, Switzerland.; 3 Laboratory of Cell and Molecular Biology, Institute of Biology, University of Neuchâtel, Rue Emile Argand 11, CH-2000 Neuchâtel, Switzerland.; 4 Center for Organismal Studies (COS), University of Heidelberg, Im Neuenheimer Feld 230, 69120 Heidelberg, Germany.; 5 Department of Molecular Plant Biology, University of Lausanne, UNIL-Sorge, 1015 Lausanne, Switzerland.

## Abstract

Precise coordination between cells and tissues is essential for differential growth in plants. During lateral root formation in *Arabidopsis thaliana*, the endodermis is actively remodeled to allow outgrowth of the new organ. Here, we show that microtubule arrays facing lateral root founder cells display a higher order compared to arrays on the opposite side of the same cell, and this asymmetry is required for endodermal remodeling and lateral root initiation. We identify that MICROTUBULE ASSOCIATED PROTEIN 70-5 (MAP70-5) is necessary for the establishment of this spatially defined microtubule organization and endodermis remodeling and thus contributes to lateral root morphogenesis. We propose that MAP70-5 and cortical microtubule arrays in the endodermis integrate the mechanical signals generated by lateral root outgrowth, facilitating the channeling of organogenesis.

## Teaser

Microtubule-associated protein-assisted perception of mechanical conflicts channels lateral root development.

## INTRODUCTION

Morphogenesis in plants depends on local growth rates and growth directions. Since plant cells are confined and linked together by rigid extracellular cell walls, spatial differences in growth can generate mechanical stresses within tissues, unlike in animal systems. The mechanical tensions caused by cells pulling or pushing on their neighbors are increasingly recognized as instructive signals during development and as an important element of the feedback mechanism coupling tissue geometry to gene expression (*1*–*3*). The lattice of cortical microtubules plays important roles in translating mechanical signals during morphogenesis (*4*–*6*). Cortical microtubules align with maximal tensile stress in plant tissues (*2*, *5*) and are required for guiding the cellulose synthase complexes to deposit cellulose microfibrils in the cell wall (*7*, *8*). Therefore, they are important regulators of anisotropic growth at the crossroads of biochemical and mechanical growth control. These conclusions were drawn from the analysis of the epidermal surface of the shoot apical meristem, where cells are under strong tension and not fully differentiated. However, it remains poorly understood how plant cells detect and integrate mechanical signals during de novo morphogenesis within a tissue.

In *Arabidopsis thaliana* (Arabidopsis), one example of morphogenesis that entails a difference in growth within a tissue is the formation of lateral root primordia (LRP) that initiate deep within the primary root, in the cell file adjacent to the xylem, the xylem pole pericycle (*9*, *10*). In response to auxin, lateral root founder cells swell, their nuclei migrate toward each other, and they divide asymmetrically to form a stage I LRP (*11*–*13*). The endodermis that overlies the forming LRP accommodates the radially expanding lateral root founder cells through a change in cell shape and/or volume loss, which requires Aux/indole-3-acetic acid (IAA) SHORT HYPOCOTYL 2 (SHY2)–mediated endodermal auxin signaling. Interference with this step results in a complete block of LRP formation (*14*) and the absence of endodermal remodeling. Several lines of evidence support a key role for the remodeling of the endodermis for lateral root initiation and morphogenesis (*14*–*16*), but the nature of the signal perceived by the endodermis upon radial expansion of the LRP remains unknown. Here, we investigate the role of cortical microtubules in the endodermis during LRP formation. We show that the endodermal cortical microtubule lattice organization and response are polarized. On the inner side, in contact with the pericycle, the arrays are more ordered than those on the outer side of the same cell. Specific disruption of microtubules in endodermal cells overlying an LRP results in delayed cellular remodeling and a flattened LRP with atypical cell division patterns. Reorganization of endodermal cortical microtubules depends on both the swelling of the underlying pericycle and an SHY2-dependent auxin response. We identify MICROTUBULE ASSOCIATED PROTEIN 70-5 (MAP70-5) as required for the organization of the endodermal cortical microtubule lattice, the remodeling of the endodermis, and the morphogenesis of the LRP. We propose that cortical microtubules and MAP70-5 contribute to the perception of LRP outgrowth by the endodermis and together function as an auxin-regulated integrator of mechanical constraints during organogenesis.

## RESULTS

### Endodermal cortical microtubules reorganize during spatial accommodation

**Fig. 1.**
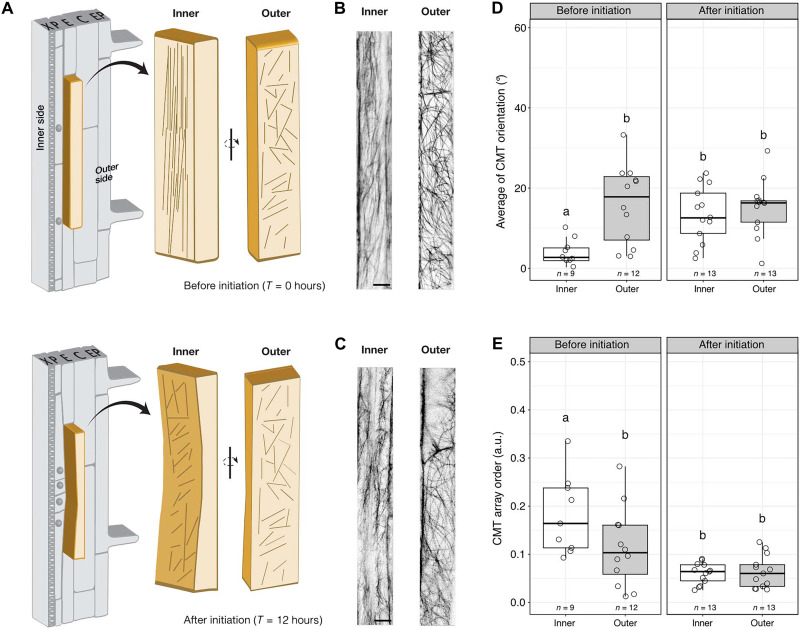
Spatiotemporally regulated cortical microtubule reorganization in overlying endodermal cells during LRP initiation. (**A**) Schematic representation of the remodeling of a differentiated endodermal cell (light brown) overlying lateral root founder cell founder cells as they radially expand and divide. The inner and outer sides of the cell remodel differently as the LRP grows (top versus bottom). Cortical microtubule (CMT) organization is simplified by lines. (**B** and **C**) Maximum projections of confocal microscopy z-stacks of *CASP1pro::mVenus:MBD* showing the organization of CMT arrays on the inner and outer side before (*T* = 0) (B) and after (*T* = 12 hours) (C) gravistimulation. (**D** and **E**) Distribution of CMT orientation before and after LRP initiation. (D) Depicts the CMT orientation in degrees with respect to the long axis of the cell, and (E) describes the CMT organization on the inner and outer side at indicated time points [arbitrary units (a.u.) 0 and 1, 0 = no order and 1 = order]. The number of endodermal cells measured is indicated (*n*). X, protoxylem; P, xylem pole pericycle; E, endodermis; C, cortex; and Ep, epidermis. Scale bars, 10 μm. Comparison between samples was performed using two-way analysis of variance (ANOVA) and Tukey’s post hoc test. Samples with identical letters do not significantly differ (α = 0.05).

To observe and quantify cortical microtubule organization and dynamics in the endodermis during LRP initiation, we generated plants expressing a fluorescent microtubule marker, *CASP1pro::mVenus:MBD*. Endodermal cells are highly polarized along the radial axis with two distinct domains, an inner and an outer side, separated by the Casparian strip (*17*). The inner side of the endodermis is in contact with the radially expanding LRP (Fig. 1A and fig. S1). Before noticeable endodermal thinning that is required to accommodate LRP development, we observed that cortical microtubules are differentially organized between the inner and outer side of differentiated endodermal cells. Cortical microtubules on the inner side form anisotropic arrays oriented along the shoot-root axis, whereas they are more isotropic on the outer side (Fig. 1, B and D). As the LRP radially expands, the cortical microtubule arrays on the inner side reorient and become more isotropic, resembling the cortical microtubule organization on the outer side (Fig. 1, C and D). Together, these results indicate that two spatially defined domains of cortical microtubule organization exist in differentiated endodermal cells and that endodermal cortical microtubule arrays facing the LRP reorganize in response to the radial expansion of the lateral root founder cells during lateral root initiation.

### Endodermal thinning and LRP morphogenesis require cortical microtubule reorganization

**Fig. 2.**
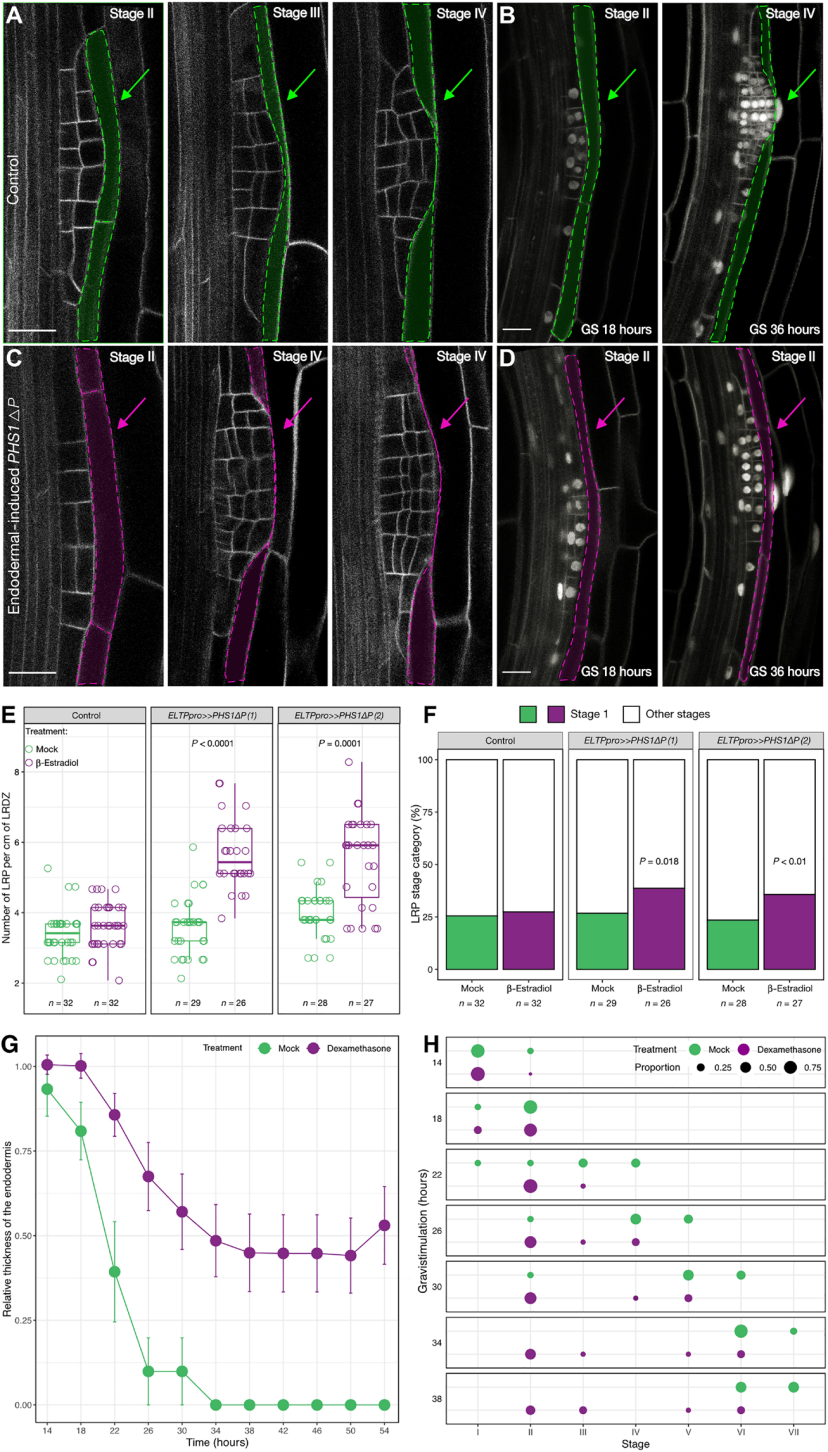
The microtubule cytoskeleton of the endodermis is required for LRP development. (**A** and **C**) LRP morphology in *ELTPpro>>PHS1*Δ*P* lines visualized by the plasma membrane marker *UBQ10pro::EYFP:NPSN12* (gray) or *CASP1pro>>PHS1*Δ*P* lines carrying the fluorescent markers *UBQ10pro::GFPx3:PIP1;4*; *GATA23pro:H2B:3xmCherry*; *DR5v2pro::3xYFP:NLS*; *RPS5Apro::tdTomato:NLS* (sC111) (*13*) (**B** and **D**) under mock (A and B) or after induction (C and D). Only the *DR5v2pro::3xYFP:NLS* and *UBQ10pro::GFPx3:PIP1;4* are visible. (B and D) Gravistimulation (GS)–induced LRP formation observed at 18 and 36 hours after GS. Scale bars, 20 μm. (A to D) The contour of the endodermal cells overlying the LRP is highlighted in green (A and B) under control conditions and pink (C and D) when PHS1ΔP was induced. (**E**) LRP density in the LR developmental zone (LRDZ). For each line, Wilcoxon rank sum test was used to compare LRP densities under the two treatments. (**F**) Analysis of stage I LRP upon disruption of the CMT. Pearson’s chi-square test with Yates’ continuity correction was used to assess whether LRP distribution is independent of CMT condition. (**G**) Quantification of relative thickness of endodermis. (**H**) Distribution of LRP developmental stages induced by GS upon induction of *CASP1pro>>PHS1*Δ*P* expression and in mock. (G and H) *n* = 59 seedlings for mock and *n* = 91 for Dex conditions.

To test whether cortical microtubule organization of endodermal cells contributes to the spatial accommodation of the LRP, we took advantage of a truncated version of PROPYZAMIDE-HYPERSENSITIVE 1 (PHS1), *PHS1*∆*P*, to interfere with the microtubule organization. Inducible, ectopic expression of *PHS1*∆*P* in cells results in depolymerization of the microtubules (*13*, *18*). We expressed *PHS1*∆*P* using two complementary endodermis-specific promoters and induction systems: *ELTPpro>>PHS1*∆*P* and *CASP1pro>>PHS1*∆*P*. Whereas *ELTPpro* is moderately expressed throughout the whole differentiated endodermis, *CASP1pro* activity peaks early in differentiating endodermal cells during Casparian strip formation (*19*, *20*). This allowed us to analyze the contributions of microtubules during early and later stages of LRP development and branching in general. We verified that expression of *PHS1*∆*P* in the endodermis results in the depolymerization of microtubules exclusively in these cells (fig. S2). With either expression system, we observed that, upon *PHS1*∆*P* induction, thinning of endodermal cells is delayed and LRP morphology is altered (Fig. 2, A to D). We quantified the impact of perturbing microtubules in the endodermis on LRP development using two different approaches. First, we compared the distribution of LRP developmental stages in the lateral root development zone, the area encompassing the first visible stage I LRP closest to the root tip and the first emerged lateral root (stage VIII) (*21*). *ELTPpro>>PHS1*∆*P*-induced microtubule depolymerization resulted in an increased number of LRPs, particularly stage I LRPs (Fig. 2, E and F). Second, we used gravistimulation-mediated lateral root formation to quantify differences in the progression of LRP development (*22*, *23*). This revealed that *CASP1pro>>PHS1*∆*P*-induced microtubule depolymerization results in a delay of LRP development (Fig. 2G and fig. S3). Thirty-six hours after induction of LRP formation by gravistimulation, LRPs are still at stages II/III, while mock-treated samples display LRPs at stages VI/VII (Fig. 2H). Together, these results show that depolymerization of microtubules in the endodermis prevents its normal remodeling, alters the cell division pattern in the LRP, and delays emergence.

### Endodermal cortical microtubule reorganization depends on Aux/IAA-mediated auxin signaling

**Fig. 3.**
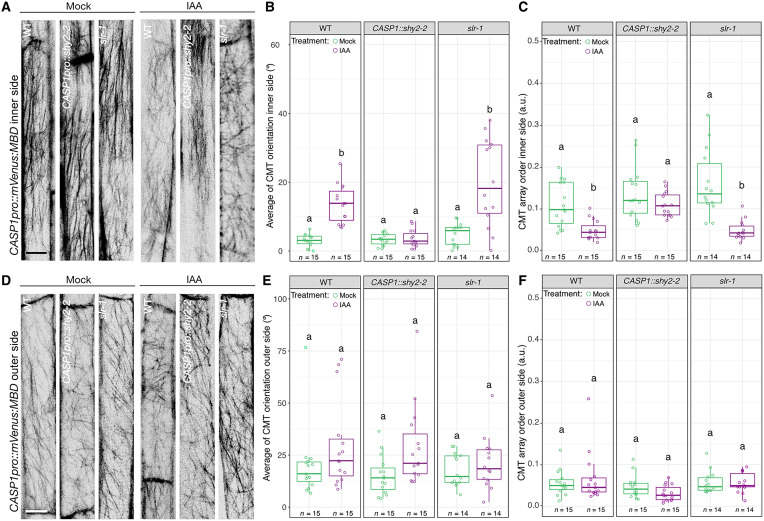
SHY2-mediated local reorganization of cortical microtubules in the endodermis. (**A**) Maximum projections of confocal z-stacks showing CMT arrays on the inner side of an differentiated endodermis cell in WT, *CASP1pro::shy2-2*, and *slr-1* plants expressing the *CASP1pro::mVenus:MBD* reporter after 24 hours of dimethyl sulfoxide (DMSO) or 1 μM indole-3-acetic acid (IAA) treatment. (**B**) Quantification of CMT orientation (0° to 90°, in respect to the long axis of the cell) and (**C**) isotropy (a.u., 0 and 1, 0 = no order and 1 = order) on the inner side of endodermal cells after 24 hours of DMSO or IAA (1 μM) treatment. (**D**) Maximum projections of confocal z-stacks showing the organization of CMT arrays on the outer side of an endodermal cell in WT, *CASP1pro::shy2-2*, and *slr-1* plants expressing the *CASP1pro::mVenus:MBD* reporter after 24 hours of DMSO or IAA (1 μM) treatment. (**E**) Quantification of CMT orientation (0° to 90°, respective to the long axis of the cell) and (**F**) isotropy (a.u., 0 and 1, 0 = no order and 1 = order) on the outer side of endodermal cells after 24 hours of DMSO or IAA (1 μM) treatment. Comparison between samples was performed using two-way ANOVA and post hoc multiple comparisons with Tukey’s post hoc test. Samples with identical letters do not significantly differ (α = 0.05). Scale bars, 10 μm.

*SHY2*-mediated auxin signaling has been shown to control spatial accommodating responses in the endodermis required for LRP formation (*14*). Accordingly, we investigated whether endodermal, SHY2-mediated auxin signaling was also required for spatially defined cortical microtubule domains and their reorganization. We used *CASP1pro::mVenus:MBD* to quantify the response of the endodermal cortical microtubule lattice in plants expressing *shy2-2*, a dominant transcriptional repressor of auxin signaling, in the differentiated endodermis (*CASP1pro::shy2-2*) (*14*). Since LRP formation is blocked in *CASP1pro::shy2-2* mutants, they were treated with IAA (1 μM) to test whether auxin could affect cortical microtubule organization in the endodermis. In wild type (WT), 1 μM IAA treatment induces a reorientation of the cortical microtubule on the inner side of endodermal cells comparable to that observed upon lateral root formation. In *CASP1pro::shy2-2* roots, cortical microtubules on the inner side do not reorganize and remain in the same organization as before auxin treatment (Fig. 3, A and B). To verify that this effect is specific to SHY2-mediated auxin signaling in the endodermis and not just due to an absence of LRP formation, we repeated the experiment in the *slr-1* mutant in which LRP initiation is also blocked, but the SHY2 response in the endodermis is unaffected (*24*). The reorganization of the cortical microtubules induced by auxin in *slr-1* expressing *CASP1pro::mVenus:MBD* is similar to that observed in WT roots (Fig. 3C), establishing that at least SHY2-mediated auxin signaling could be responsible for the rearrangement of cortical microtubules on the inner side of endodermal cells rather than an indirect consequence of auxin-induced lateral root development. The reorganization of cortical microtubules on the outer side of WT, *CASP1pro::shy2-2*, and *slr-1* is not affected (Fig. 3, C to F). Consequently, this suggests that at least SHY2-mediated auxin signaling is required for the spatially defined remodeling of cortical microtubules in the endodermis.

### Aux/IAA proteins regulate MAP70-5 in accommodating endodermal cells

**Fig. 4.**
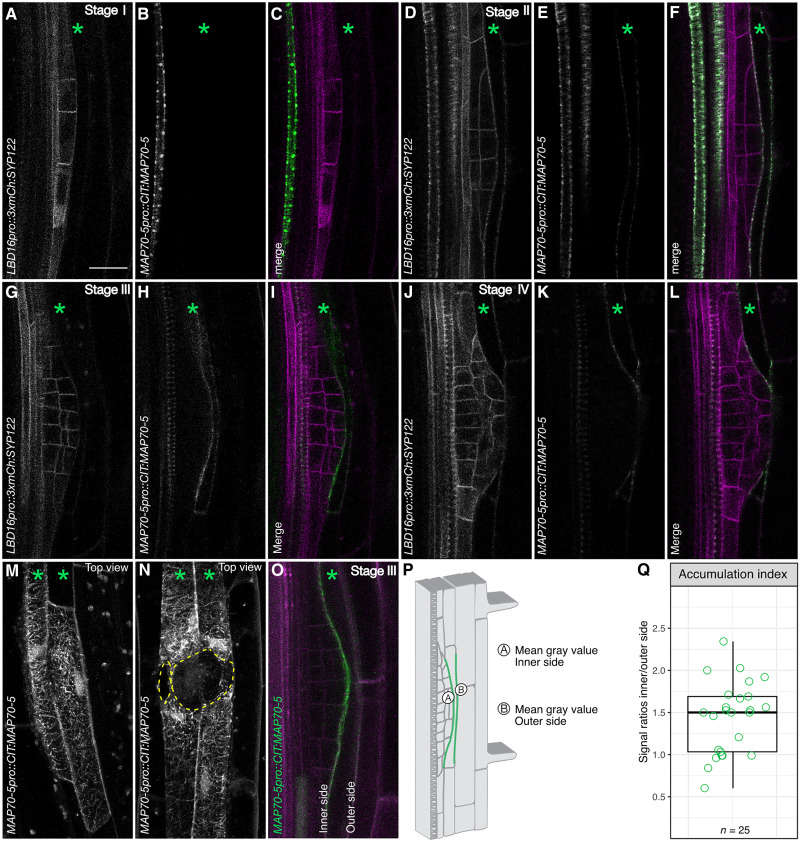
MAP70-5 expression in the endodermis correlates with spatial accommodation. (**A** to **O**) Confocal images of developing lateral roots in seedlings expressing *LBD16pro::3xmCherry:SYP122* [plasma membrane, gray (A, D, G, and M) or magenta (C, F, I, and L)] to visualize the LRP and *MAP70-5pro::CITRINE:MAP70-5* (gray: B, E, H, K, M, and N or green: C, F, I, and L). (A to C) Stage I: *CITRINE:MAP70-5* is expressed in a differentiating metaxylem cell. (D to L) *CITRINE:MAP70-5* is induced in endodermal cells overlying the LRP from stages II to IV. (A to L) Images are single confocal planes. (M and N) Maximum projections of confocal image stacks depicting a surface view of endodermal cells overlying the LRP: (M) stages IV and (N) V. (O) Single confocal image showing accumulation of CITRINE:MAP70-5 on the inner side of an endodermal cell overlying a stage IV LRP. (A to L and O) Images of single confocal sections. (M and N) Maximum projections of z-stacks. Green asterisks indicate endodermal cell files. Yellow dashed circles indicate the area of the LRP. (O) Stage III LRP showing accumulation of CITRINE:MAP70-5 (green) on the inner side of an overlying endodermal cell. Magenta shows plasma membrane labeled by *UBQ10pro::RCI2a:tdTomato* (*46*). (**P**) Schematic representation of inner and outer domains used for quantification of the accumulation index of CITRINE:MAP70-5 in endodermal cells overlying LRP (stages II to IV). (**Q**) Quantification of the mean gray values of the inner and outer domains. For the accumulation index, the collected data were normalized, and the ratio between the inner and outer domains was calculated. Scale bar, 20 μm.

We next mined an auxin-induced transcriptome of differentiated endodermal cells for cytoskeleton-related genes that could regulate cortical microtubule organization and/or dynamics (*24*). We identified *MAP70-5* as a potential Aux/IAA-dependent regulator of cortical microtubule organization in the endodermis. Plants expressing a *MAP70-5pro::CITRINE:MAP70-5* fusion (*CITRINE:MAP70-5*) display a spiral or punctate localization pattern in differentiating proto- and metaxylem cells, respectively (Fig. 4, A to C). This is in agreement with a proposed function of MAP70-5 in regulating secondary cell wall formation during xylem formation (fig. S4 and movie S1) (*25*, *26*). We also observed *CITRINE:MAP70-5* expression in the pericycle and endodermis in the early differentiating zone of Arabidopsis roots, the area where lateral root founder cell specification is reported to occur (fig. S4) (*27*). This early expression in the pericycle and endodermis of *MAP70-5:CITRINE* appeared not to be affected in *CASP1pro::shy2-2* roots (fig. S4). During LRP formation, we observed induction of *CITRINE:MAP70-5* specifically in endodermal cells overlying the growing LRP from stage II onward (Fig. 4, D to L, and movie S2). *MAP70-5* contains a predicted auxin-responsive element (TGTCTC) (*28*, *29*) in its promoter region, in line with auxin-dependent regulation. Auxin treatment induces an expansion of the expression domain of *CITRINE:MAP70-*5 in endodermal and cortex cells throughout the root (fig. S5, A to C). This auxin-mediated induction of *CITRINE:MAP70-5* is completely blocked in *CASP1pro::shy2-2* seedlings (fig. S5, D to F). From this, we determine that, while endodermal expression of MAP70-5 can occur independently of auxin activity, the specific induction of *MAP70-5* in endodermal cells overlying the LRP is dependent on *Aux/IAA*-mediated auxin signaling. Induction of *CITRINE:MAP70-5* in the cortex of *CASP1pro::shy2-2* roots is also blocked, suggesting that this also requires endodermis-mediated auxin signaling. In endodermal cells overlying an LRP, CITRINE:MAP70-5 localizes to filamentous structures in the cell periphery. Four-dimensional live-cell imaging of the endodermis during LRP formation revealed that CITRINE:MAP70-5 labels dynamic structures, and their organization appears to become more isotropic when the LRP reaches stage IV; the point at which it traverses the endodermis (Fig. 4, N and O, and movie S3). We also observed that CITRINE:MAP70-5 displays a differential localization pattern in the accommodating endodermal cells (Fig. 4, O to Q). Quantification of signal intensity on the inner and outer side of these cells reveals an enrichment of CITRINE:MAP70-5 (*r* = 1.44 ± 0.428, *n* = 25) on the inner side of endodermal cells overlying stages III/IV LRPs (Fig. 4P). Imaging of CITRINE:MAP70-5 and a microtubule marker (*ELTPpro::mScarlet-I:MBD*) revealed a partial colocalization between MAP70-5 and cortical microtubules (fig. S6, A to F). Depolymerization of microtubules, either by oryzalin or via induction of *PHS1*Δ*P* expression in the endodermis (*ELTPpro>>PHS1*∆*P*), revealed that, in both cases, the localization of CITRINE:MAP70-5 gradually shifted from a filamentous to a more diffuse cytosolic localization (fig. S6, G to L). Together, these results show that MAP70-5 partially colocalizes with cortical microtubules in endodermal cells and requires intact microtubules for its localization. Furthermore, CITRINE:MAP70-5 preferentially accumulates on the inner side of endodermal cells overlying stage III/IV LRPs, alluding to a potential contribution to the reorganization of cortical microtubules during spatial accommodation.

### MAP70-5 affects LRP formation in a noncell autonomous manner

**Fig. 5.**
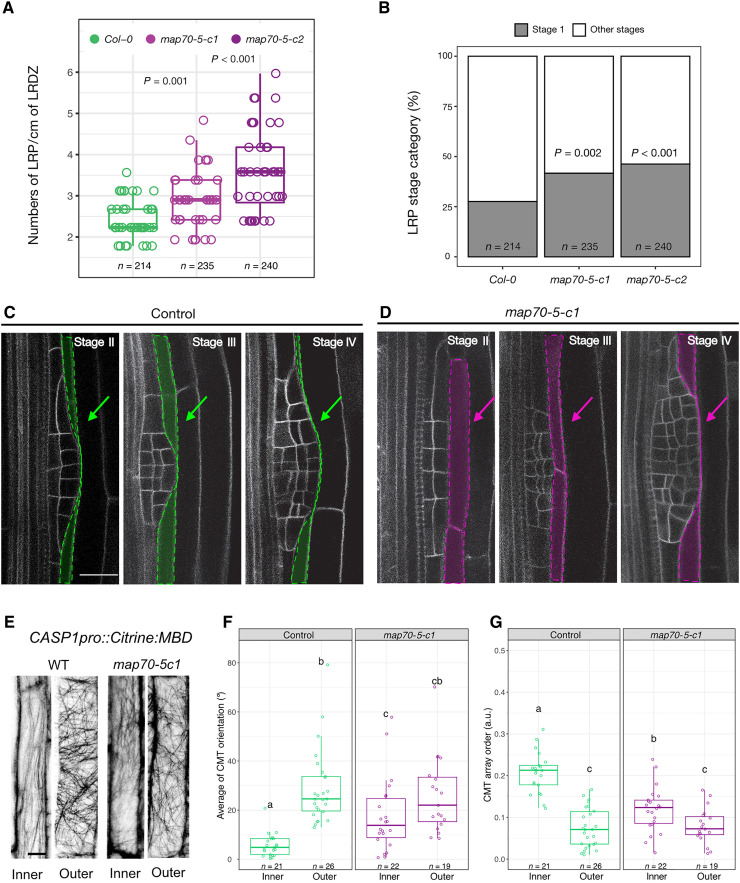
MAP70-5 is required for spatial accommodation responses in the endodermis. (**A**) lateral root density in the LRDZ of WT compared to *map70-5-c1* and *map70-5-c2*. For each line, Wilcoxon rank sum test with continuity correction was performed to compare LR densities. (**B**) Staging of LRs reveals an accumulation of stage I LRP in the *map70-5-c1* and *map70-5-c2* compared to WT roots. Pearson’s chi-square test with Yates’ continuity correction was used to assess whether LRP distribution is independent in the *map70-5* mutants. (**C** and **D**) Single confocal planes showing LRP morphology in WT and *map70-5-c1* through stages I to IV visualized by the plasma membrane marker *UBQ10pro::EYFP:NPSN12*. Contours of endodermal cells overlying the LRP are highlighted in green (C) under control conditions and pink (D) when PHS1ΔP was induced. (**E**) Maximum projection of confocal z-stacks showing CMTs on the inner and outer side of WT and *map70-5-c1* endodermal cells. (**F**) CMT orientation (0° to 90°, respective to the long axis of the cell). (**G**) CMT isotropy on the inner and outer side (a.u., 0 = no order and 1 = order). Scale bars, 10 μm. Comparison between samples was performed using two-way ANOVA and post hoc multiple comparisons with Tukey’s test. Samples with identical letters do not significantly differ (α = 0.05).

To test whether MAP70-5 is required for reorganization of cortical microtubules during the remodeling of the endodermis throughout LRP formation, we generated *map70-5* mutants, *map70-5-c1* and *map70-5-c2*, which both contain large deletions, resulting in an early stop codon (fig. S6). Both *map70-5* alleles show a reduction in primary root length and have a smaller lateral root developmental zone (LRDZ) (fig. S7, B to D). Quantification of the distribution of LRP stages along the LRDZ of both *map70-5* alleles revealed a significant increase in total LRPs (Fig. 5A), with both alleles showing a higher proportion of stage I LRPs (Fig. 5B), suggesting an increased rate of lateral root initiation and a delay in emergence. This was confirmed by introgression of the plasma membrane marker *UBQ10pro::EYFP:NPSN12* into *map70-5-c1* and comparison of LRP morphology to WT plants (stages I to IV). In contrast to WT, *map70-5-c1* mutants display flattened LRPs and a turgid endodermis from stage II onward (Fig. 5, C and D, and table S1). This phenotype is similar to the one observed upon depolymerization of endodermal microtubules (Fig. 2). We then set out to test whether MAP70-5 plays a role in the organization and dynamics of the endodermal cortical microtubule lattice during LR formation. Using *CASP1pro::mVenus:MBD* to quantify the organization of the cortical microtubules in endodermal cells revealed that the *map70-5-c1* mutant has lost the differential organization of the cortical microtubule lattice on the inner side of the cell. In *map70-5-c1*, the organization of cortical microtubules in the endodermis is comparable to that observed in endodermal cells before lateral root initiation (Figs. 2 and 5, E to G), indicating that MAP70-5 is required for the spatial reorganization of cortical microtubule arrays in the endodermis. Together, these results show that MAP70-5 acts during two phases of spatial accommodation by the endodermis during LRP development. In the first phase, it functions as a negative regulator that confines LRP initiation via cortical microtubule array organization on the inner side of endodermal cells. Interfering with this role will thus result in more early stage LRP. In the second phase, MAP70-5 appears to act as a positive regulator of LRP development and morphogenesis, regulating the spatial accommodation by the endodermis. Interfering with this function will thus delay the growth of the LRP through the endodermis, resulting in an increased number of LRP with an atypical morphogenesis. We propose that MAP70-5 integrates and possibly relays instructive signals via the cortical microtubules in the endodermis during lateral root formation.

## DISCUSSION

Plant development, just like in other organisms, relies on the integration of both chemical and physical signals (*30*–*32*). In contrast to what we have learned about this process in surface cell layers (*33*–*35*), it is still not clear how mechanical conflicts generated during differential growth in deep lying plant tissues are integrated and translated into a developmental output, and whether similar mechanisms are at play. Previously, we showed that asymmetric cortical microtubule organization of LR founder cells is required for their asymmetric expansion and to license LRP initiation. Moreover, we showed that endodermal auxin signaling was required for the remodeling of the lateral root founder cells. However, how the endodermis contributed to LRP initiation remained unknown (*13*).

Here, we identify a molecular mechanism operating in the endodermis that is required to channel organ initiation and development in the xylem pole pericycle. By combining live-cell imaging and cell type–specific genetic perturbations of microtubule organization, we show that cortical microtubules in the endodermis are required for endodermal remodeling and normal LRP development. Cortical microtubule arrays on the side of endodermal cells facing the lateral root founder cells are more anisotropic than those on the other side of the same cell, and this is required for proper LRP morphogenesis. To accommodate the newly formed organ, the cortical microtubules on the inner side of overlying endodermal cells will subsequently become more isotropic to facilitate thinning of endodermal cells. However, the observation that depolymerization of endodermal microtubules, which is assumed to make cells less mechanically resistant, results in an increase in stage I LRPs and interferes with endodermal thinning (Fig. 2). This rather suggests that a tight regulation of endodermal microtubule organization is required to coordinate the expansion growth of the LRP. Initially, anisotropic cortical microtubule arrays on the inner side of overlying endodermal cells are required to suppress lateral root initiation, possibly by providing mechanical feedback. After initiation, the endodermal cortical microtubule arrays need to become more isotropic to channel the outgrowth of the LRP. Thus, a regulated switch from anisotropic to more isotropic endodermal cortical microtubule arrays is necessary for both the perception of a signal instructing the endodermis to remodel to initiate and for the completion of this developmental program, and both processes require a SHY2-dependent auxin signaling module.

Analyzing the Aux/IAA-dependent transcriptome of endodermal cells (*24*), we identified MAP70-5 as a candidate regulator of cortical microtubule organization during spatial accommodation. CITRINE:MAP70-5 is induced in overlying endodermal cells and partially colocalizes with cortical microtubules, whereas *map70-5* mutants show an increase in stage I LRP and altered morphogenesis up to stage IV LRP (Fig. 5). Endodermis-specific microtubule depolymerization and the *map70-5* mutants have similar phenotypes, suggesting that MAP70-5 is an important relay in the communication between the LRP and the endodermis. In *map70-5* mutants, the endodermis is predicted to be altered in its ability to perceive or respond to the outgrowth of the LRP. Quantification of the cortical microtubule array order on the inner and outer side of endodermal cells revealed that MAP70-5 also functions before lateral root formation. Endodermal cells in roots of *map70-5* mutants no longer display the spatially defined, differential cortical microtubule array order (Fig. 5, E to G). It appears that cortical microtubule arrays on the inner side are already in a configuration that facilitates lateral root initiation. A plausible mechanism is that MAP70-5 locally regulates cortical microtubule array order on the inner side of the endodermis via bundling, and this is a part of a mechanism that spatially restricts LRP initiation. Subsequently, MAP70-5 is required for proper endodermal thinning to properly channel LRP organogenesis, since *map70-5* mutants also display a significant increase in flattened LRP, which we also observed upon depolymerization of endodermal microtubules. This is again in support that microtubule organization needs to be tightly regulated to accommodate organ growth.

How might MAP70-5 regulate the cell shape of endodermal cells overlying LR founder cells? Since these endodermal cells are fully differentiated, it is unlikely that the observed effects are due to defects in the guidance or dynamics of the cell wall synthesis machinery. The fact that endodermal cells of *map70-5-c1* roots have lost the differentially organized cortical microtubule array order on the inner side would support a direct role for the microtubule cytoskeleton itself during founder cell–endodermis communication and possibly also during LRP outgrowth. Moreover, the punctate localization of CITRINE:MAP70-5 at the cell periphery suggests that MAP70-5 might be partially localized at the plasma membrane. Since the overlying endodermal cells must undergo a marked change in cell volume to accommodate the outgrowth of the LRP, we hypothesize that MAP70-5 is required to transduce the mechanical constraints detected at the interface between the LRP and neighboring endodermal cells and to regulate cortical microtubule array order to channel organogenesis. Thus, we propose a role for MAP70-5 as an integrator of mechanical constraints generated during the reestablishment of three-dimensional differential growth within a tissue.

## MATERIALS AND METHODS

### Plant materials and manipulation

The *A. thaliana* Columbia ecotype (Col-0) was used. Seeds were surface sterilized (5% sodium hypochlorite and 0.01% Tween 20 or 70% ethanol and 0.1% SDS) and placed on ½ Murashige and Skoog medium containing 1% agar (AppliChem or Duchefa). Following stratification (4°C in the dark, *t* > 24 hours), seedlings were grown at 22°C vertically under constant light or long-day conditions (16-hour light/8-hour dark). *Agrobacterium tumefaciens* (GV3110)–based plant transformation was carried out using the floral dip method (*36*). All plant lines examined were homozygous if not indicated otherwise. Homozygosity was either determined upon the presence of the Fast Red cassette, by antibiotic resistance, verification of the fluorescent fusion proteins at the microscope, and/or polymerase chain reaction. Besides the below mentioned created vectors and plants lines, this study uses the triple marker line *UBQ10pro::GFP:PIP1;4 x GATA23pro::H2B:mCHERRY x DR5v2pro::NLS-3xYFP* sC111 (*13*) and the membrane marker line *UBQ10pro::EYFP:NPSN12* (*37*). For experiments with inducible gene expression and/or drug treatments, β-estradiol (β-est), dexamethasone (Dex), and oryzalin stocks were dissolved in EtOH, dimethyl sulfoxide (DMSO), or water, used as indicated.

### Construction of vectors and transformation

To generate *ELTPpro>>PHS1*∆*P* (*pFR-ELTPpro-XVE>>PHS1*∆*P*), *pEN-4_ELTPpro-XVE_1R*, *pEN-1_PHS1*∆*P_2*, and *pEN-2r_mCherry_3* were recombined into pFastRed-3xGW (*38*). To generate *CASP1pro>>PHS1*∆*P* (*CASP1pro::LhG4-GR-6xOP:PHS1*∆*P-mCherry*), we used GreenGate assembly (*39*) to combine *pGGM-CASP1pro::LhG4-GR* (*40*) with *pGGN-6xOP:PHS1*∆*P-mCherry-FastRed* (*13*). To generate *pGr179-CASP1pro::mVENUS:MBD*, KpnI was used to exchange *XPPpro* with *CASP1pro* in *pGr179-XPPpro::mVENUS:MBD* (*13*) from *CASP1pro::CITRINE:SYP122* (*14*) using *Kpn*I. To generate *pGr179-LBD16pro::3xmCherry:SYP122*, KpnI was used to exchange *XPPpro* with *LBD16pro* in *pGr179-XPPpro::3xmCherry:SYP122* (*38*). To generate pUC57-L4_MAP70-5pro_R1, 1573 bp of upstream sequence of MAP70-5 (AT4G17220) was amplified and cloned into *Kpn*I-digested pUC57-L4_KpnI/XmaI_R1. For pEN-2R_MAP70-5_3, the genomic sequence of *MAP70-5* (At4g17220) was amplified and recombined into pENTR1-2 using BP Clonase II (www.thermofisher.com). To assemble *MAP70-5pro:CITRINE:MAP70-5*, pUC57-L4_MAP70-5pro_R1, pEN-1_CITRINE_2, and pEN-2R_MAP70-5_3 were recombined into pH7m34GW,0. To generate *pFR-ELTPpro::mScarlet-I:MBD*, pEN-4_ELTPpro_R1, pEN-1_mScarlet-I_2, and pEN-2R_MBD_3 were recombined into pFastRed-3xGW. pEN-1_mScarlet-I_2 was amplified and recombined into pENTR1-2, and pEN-2R_MBD_3 was synthesized (www.thermofisher.com). Expression vectors were assembled using MultiSite Gateway Cloning with LR Clonase II Plus (www.thermofisher.com). For CRISPR-Cas9–mediated generation of MAP70-5 mutants, we used pFR-UBQ-CAS9-1xGW (*41*). Three different single guide RNA (sgRNA) constructs were cloned, targeting different areas of MAP70-5, using a combination of GreenGate (*39*) and Gateway cloning. Primers for amplifying fragments used for cloning and sgRNAs used for generating *map70-5-c1* and *map70-5-c2* mutants are shown in table S2 and fig. S7. In addition to the deletion shown in fig. S6, *map70-5-c1* also has an insertion of a single A after position 1774 at the target site of sgRNA3-1. This is downstream of the early stop codon introduced by the large deletion. For transient expression in *Nicotiana benthamiana*, *pFR-35Spro::mScarlet-I:MBD*, *pFR-35Spro::mScarlet-I:MAP70-5*, *pFR-35Spro::mCITRINE:map70-5c1*, and *pFR-35Spro::mCITRINE:map70-5c2* were generated, and plants were infiltrated as described (*42*). The genomic sequence of *map70-5-c1* and *map70-5c2* was amplified and recombined into pDONR1-2. Resulting pENTRY vectors were assembled into pFastRed-3xGW: *pEN-4_35spro_R1*, *pEN-1_mScarlet-I_2*, and *pEN-2R_MBD_3*; *pEN-4_35spro_R1*, *pEN-1_mScarlet-I_2*, and *pEN-2R_MAP70-5*; *pEN-4_35spro_R1*, *pEN-1_mCITRINE_2*, and *pEN-2R_map70-c1*; and *pEN-4_35spro_R1*, *pEN-1_mCITRINE_2*, and *pEN-2R_map70-c2*. 35*S* promoter–driven expression of *mCitrine:map70-5-c1* and of *mCitrine:map70-5-c2* alleles resulted only faint cytosolic fluorescence in the cytosol of infiltrated tobacco leaves (fig. S7).

### Microscopy

Live-cell imaging performed with a Leica true confocal scanner (TCS) SP8X-MP, Leica TCS SP8X, or a Leica TCS SP8 DIVE system, equipped with a resonant scanner (8 kHz), a 63×, numerical aperture (NA) = 1.2 water immersion objective; a 63×, NA = 1.3 glycerol immersion objective; a 63×, NA = 1.4 oil immersion objective; or a 40×, NA = 1.2 water immersion objective. For excitation of CITRINE, mVENUS, and mCherry, either an insight DS+ Dual ultrafast near-infrared laser or a Chameleon Vision II tuned to 960 nm was used for multiphoton excitation. For detection, non–descanned supersensitive photon counting hybrid detectors (HyD), operated in photon counting mode, were used. Enhanced yellow fluorescent protein (EYFP)/mVENUS fluorescence was filtered with a cyan FP/YFP filter cube (483/32 and 535/30) or using the 4Tune detector set to the same detection window. For colocalization of *MAP70-5pro::CITRINE:MAP70-5* and *ELTPpro::mScarlet-I:MBD*, we used a white light laser (WLL) for excitation (517 nm for citrine and 561 nm for mScarlet-I). Fluorescence was detected using HyD detectors operating in photon counting mode (527 to 560 nm for citrine and 575 to 620 nm for mScarlet-I). For cortical microtubule array observations, *CASP1pro::mVenus:MBD* was excited with a WLL at 514 nm and detected using the HyD detector in standard mode (525 to 560 nm). Images were acquired using sequential scanning mode to minimize cross-talk between different detector channels. The acquired z-stacks in this study were taken in step sizes ranging from 0.5 to 1.0 μm. For long-term imaging, time-lapse data were acquired every 15 to 30 min for up to 16 hours (movies S1 to S3).

### LRP analysis

For endodermal specific MT disruption, *ELTPpro>>PHS1*∆*P* in the *UBQ10pro::EYFP:NPSN12* background [W131Y; (*37*)] and *UBQ10pro::EYFP:NPSN12* as control lines were germinated on plates containing either 5 μM β-est or EtOH (mock). For the *CASPpro>>PHS1*∆*P* in the *UBQ10pro::GFPx3:PIP1;4* *GATA23pro:H2B:3xmCherry*; *DR5v2pro::3xYFP:NLS*; *RPS5Apro::tdTomato:NLS* (sC111) background (*13*), plants were germinated on plates for 5 days then shifted to plates containing either 10 μM Dex or EtOH (mock) and gravistimulated by rotating the plate 180°.

Phenotyping and staging along the LRDZ were performed by live-cell imaging (Leica TCS SP8X-MP). Experiments were repeated six times. To quantify LRP in the *map70-5* mutants along the LRDZ, seedlings were cleared as published (*43*) and staged using a Nikon E800 upright microscope equipped with differential interference contrast. For visualization of the cell outline, *map70-5-c1* plants were crossed with the W131Y cell membrane marker line, and homozygous F_3_ plants were imaged and staged from stage I to IV (960-nm excitation; Leica SP8-MP DIVE). To determine total root and LRDZ length of *ELTPpro>>PHS1*∆*P* in the *UBQ10pro::EYFP:NPSN12* and *map70-5* mutants, 7-day-old seedlings were scanned and either measured from the root tip to the first visible emerged LR (LRDZ) or from the root tip to the hypocotyl (total root length), using the segmented line tool in FIJI. To analyze differentially expressed CITRINE:MAP70-5 in accommodating endodermal cells overlying the LRP, a combination of natural- and gravistimulus-induced LRs were imaged (Leica TCS SP8X-MP). For the former, plants were grown for 6 days. For the latter, seedlings were turned 90° after 5 days and imaged after a further 24 hours. Using the segmented line tool in FIJI, ratios between mean intensity values of CITRINE:MAP70-5 inner and outer side were calculated, describing the accumulation index (Fig. 2, O and P).

### Image processing and assembly

The acquired microscopy images were processed with FIJI (v2.0.0-rc-59/1.51k, https://fiji.sc/) and Affinity Photo v1.6.7. Figure assembly was performed with Affinity Designer v1.83 and Adobe Illustrator 2021 (V25.0).

### Data visualization and statistics

All experiments were performed at least three times. Sample size (*n*) for each plant line and treatment are denoted in the figures. Statistical comparisons (Wilcoxon rank sum test and Pearson’s chi-square test) and box and bar plots were made with R software (*44*). Methods and *P* values are summarized in figure legends.

### IAA treatments

To examine endodermal microtubule organization upon IAA treatment in different genetic backgrounds (Fig. 3), 5-day-old seedlings were transferred to IAA containing medium (1 μM) for 21 hours and subsequently imaged (Leica TCS SP8X). To ensure that the same developmental stage was studied, only endodermal cells adjacent to the fifth cortex cell above the first differentiated protoxylem cell were imaged from the top. From this, z-stacks were generated. During imaging, the seedlings were kept in microscopy chambers and covered with a thin layer of medium either supplemented with IAA or mock. By the end of the experiment, seedlings had been exposed to IAA for 24 hours. To test whether MAP70-5 expression is dependent on auxin signaling (fig. S6), 5-day-old seedlings were transferred to IAA containing liquid medium (10 μM) and imaged after 24 hours (Leica TCS SP8 MP).

### Cortical microtubule array analysis

The Fiji plugin FibrilTool (*45*) was used to analyze cortical microtubule orientation and order in the endodermis. Z-stacks of endodermal cells expressing *CASP1pro:mVenus:MBD* were acquired (Leica TCS SP8X), either on the inner or outer side of the endodermal cell. Maximum projections of the acquired z-stacks were generated and aligned (via rotation) to the longitudinal axis of cells, resembling 0°. Regions of interest were set to analyze the cortical microtubule orientation, and absolute values (from 0° to 90°) were used to visualize the direction of cortical microtubules. The anisotropy analysis describes the microtubule organization in arbitrary units. Zero indicates isotropic (not ordered), and 1 indicates anisotropic (ordered) cortical microtubule organization.
