# Supplementary material for: Microtubule-based perception of mechanical conflicts controls plant organ morphogenesis
Source: Sci Adv. 2022 Feb 9;8(6):eabm4974. doi: 10.1126/sciadv.abm4974 (PMC8827652; doi:10.1126/sciadv.abm4974)
Supplement: Supplementary file 1 — Figs. S1 to S7 Tables S1 and S2 Legends for movies S1 to S3 [file sciadv.abm4974_sm.pdf]

Science Advances
AAAS

Supplementary Materials for

Microtubule-based perception of mechanical conflicts controls plant organ morphogenesis

Dorothee Stöckle, Blanca Jazmin Reyes-Hernández, Amaya Vilches Barro, Milica Nenadić, Zsofiá Winter, Sophie Marc-Martin, Lotte Bald, Robertas Ursache, Satoshi Fujita, Alexis Maizel*, Joop EM Vermeer*

*Corresponding author. Email: alexis.maizel@cos.uni-heidelberg.de (A.M.); josephus.vermeer@unine.ch (J.E.M.V.)

Published 9 February 2022, *Sci. Adv.* **8**, eabm4974 (2022)
DOI: 10.1126/sciadv.abm4974

The PDF file includes:

Figs. S1 to S7
Tables S1 and S2
Legends for movies S1 to S3

Other Supplementary Material for this manuscript includes the following:

Movies S1 to S3

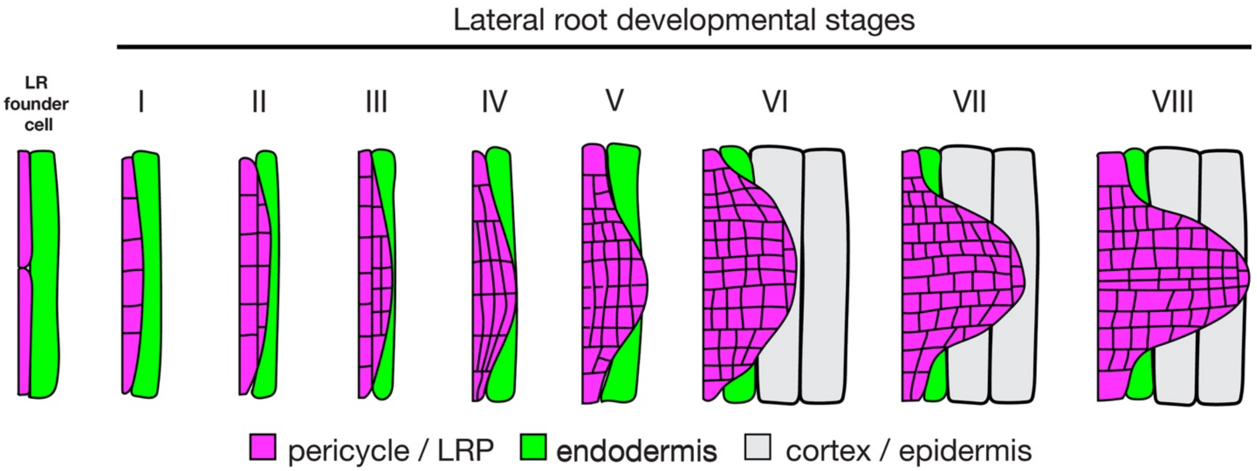

Fig. S1. Different stages of Arabidopsis lateral root development

Schematic overview of the different stages of lateral root development in Arabidopsis from lateral root founder cells until an emerged lateral root (Stage VIII). The number of each developmental stage refers to the number of cell layers in the lateral root primordium. Pericycle / lateral root primordium cells are shown in magenta, endodermis in green and cortex / epidermis in grey.

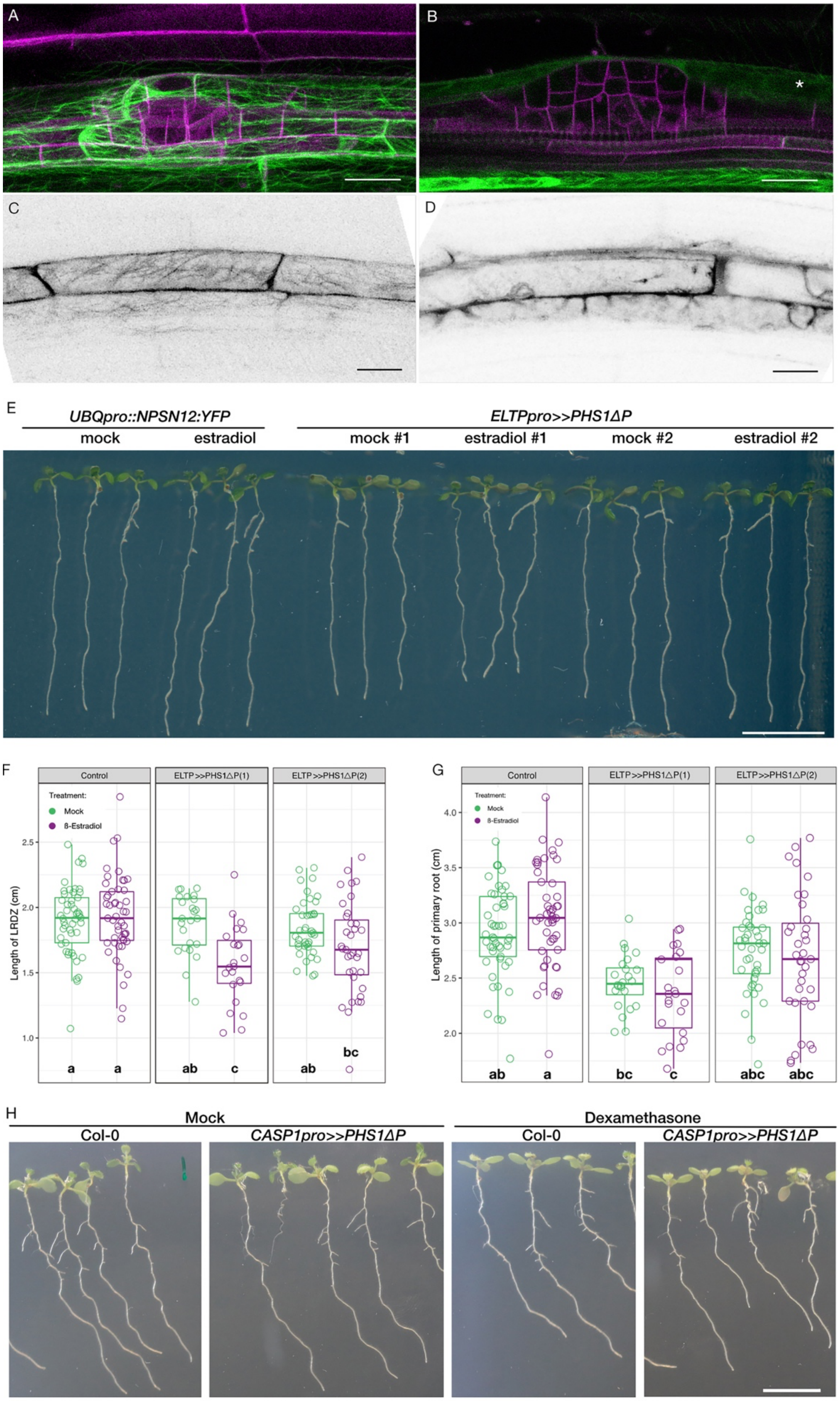

A
B
*
C
D
E
UBQpro::NPSN12:YFP
ELTPpro>>PHS1ΔP
mock
estradiol
mock #1
estradiol #1
mock #2
estradiol #2
F
Control
ELTP>>PHS1ΔP(1)
ELTP>>PHS1ΔP(2)
Treatment:
Mock
β-Estradiol
Length of LRDZ (cm)
2.5
2.0
1.5
1.0
a
a
ab
c
ab
bc
G
Control
ELTP>>PHS1ΔP(1)
ELTP>>PHS1ΔP(2)
Treatment:
Mock
β-Estradiol
Length of primary root (cm)
4.0
3.5
3.0
2.5
2.0
ab
a
bc
c
abc
abc
H
Mock
Dexamethasone
Col-0
CASP1pro>>PHS1ΔP
Col-0
CASP1pro>>PHS1ΔP

Fig. S2. Interfering with endodermal cortical microtubule organization impacts LR formation.

(**A**) *CASP1pro::mVenus:MBD* (green) labelling microtubules, and *LBD16pro::3xmCherry:SYP122* (magenta) the plasma membrane in the LRP, under mock conditions. (**B**) Disruption of the CMT in the endodermis after *ELTPpro>>PHS1ΔP* induction (5 µM ß-est, 24 h). (**C**) *CASP1pro>>PHS1ΔP* / *CASP1pro::mVenus:MBD* showing *CASP1pro::mVenus:MBD* under mock (H_2O) or (**D**) upon dexamethasone (Dex) treatment (10 µM). (**A**-**D**) Maximum projection of z-stacks. Scale bar = 20 µm. (**E**) Image of seven day old seedlings carrying *UBQ10pro::EYFP:NPSN12* and *ELTPpro-XVE>>PHS1ΔP* grown on either 5 µM β-est (estradiol) or mock (EtOH) supplemented medium. Scale bar = 1 cm. (**F**) Quantification of the LRDZ and (**G**) total root length. Statistical analysis was performed using one-way ANOVA followed by *post-hoc* multiple comparisons with Tukey's test. Samples with identical letters do not significantly differ ($\alpha = 0.05$). (**H**) Eight-day old seedlings of marker lines sC111 (Col-0) and sC111 combined with *CASP1pro>>PHS1ΔP* on mock (H_2O) or Dex-supplemented medium (10 µM).

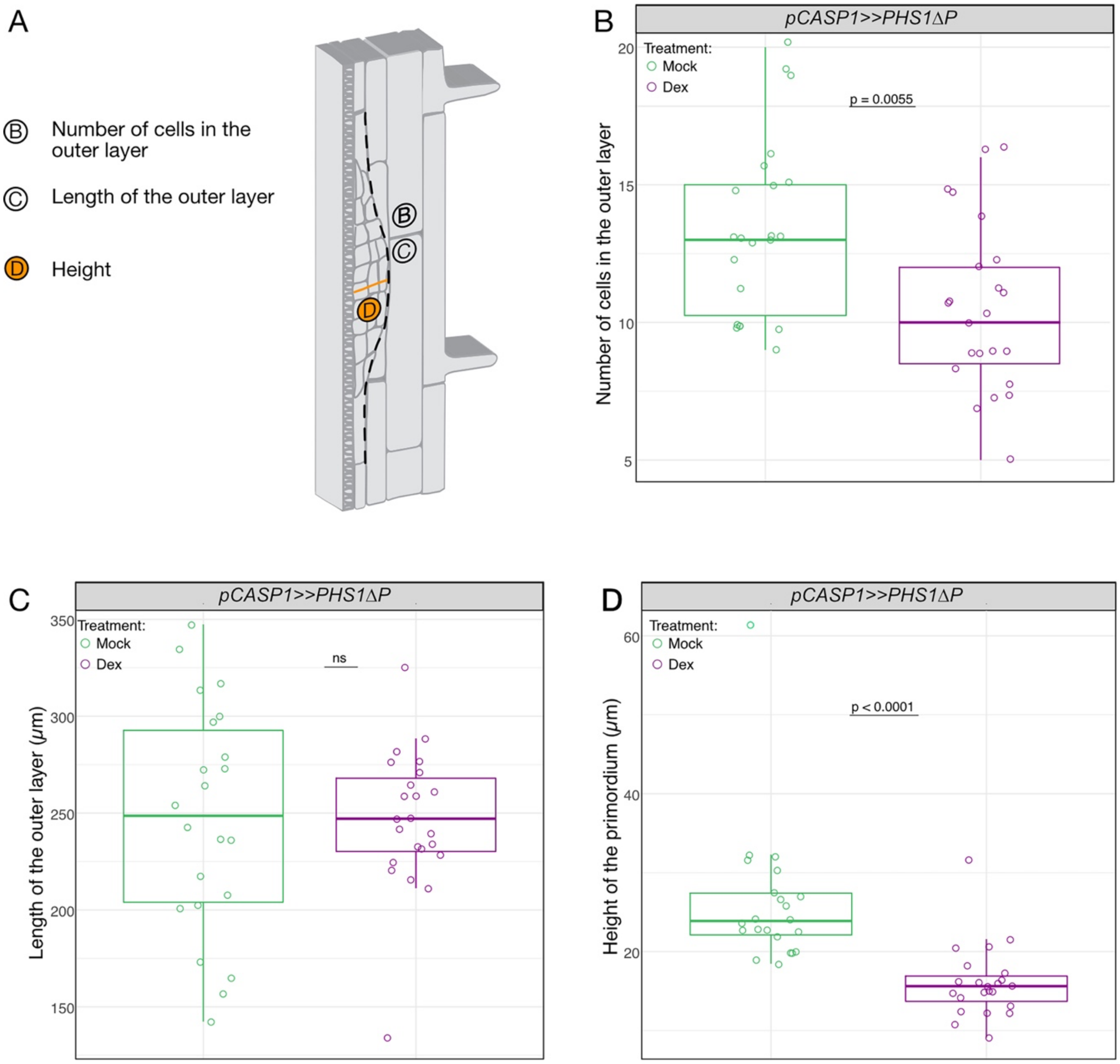

Fig. S3. Quantification of the LR morphology after 36 h gravistimulation.
(**A**) Drawing of a longitudinal root section displaying a stage III LRP. Letters in (**A**) indicate data represented in (**B-D**). (**B**) Number of cells in the outer layer of the LRP, (**C**) length of the outer layer of the LRP in µm, and (**D**) maximum height of the LRP. The dashed line flanks the outline of the LRP and displays where the measurements were taken and cells were counted. Statistical analysis of *CASP1pro>>PHS1ΔP* in the sC111 background on mock (H_2O, n = 22) and (Dex, n = 23) was performed via a Wilcoxon rank-sum test with continuity correction.

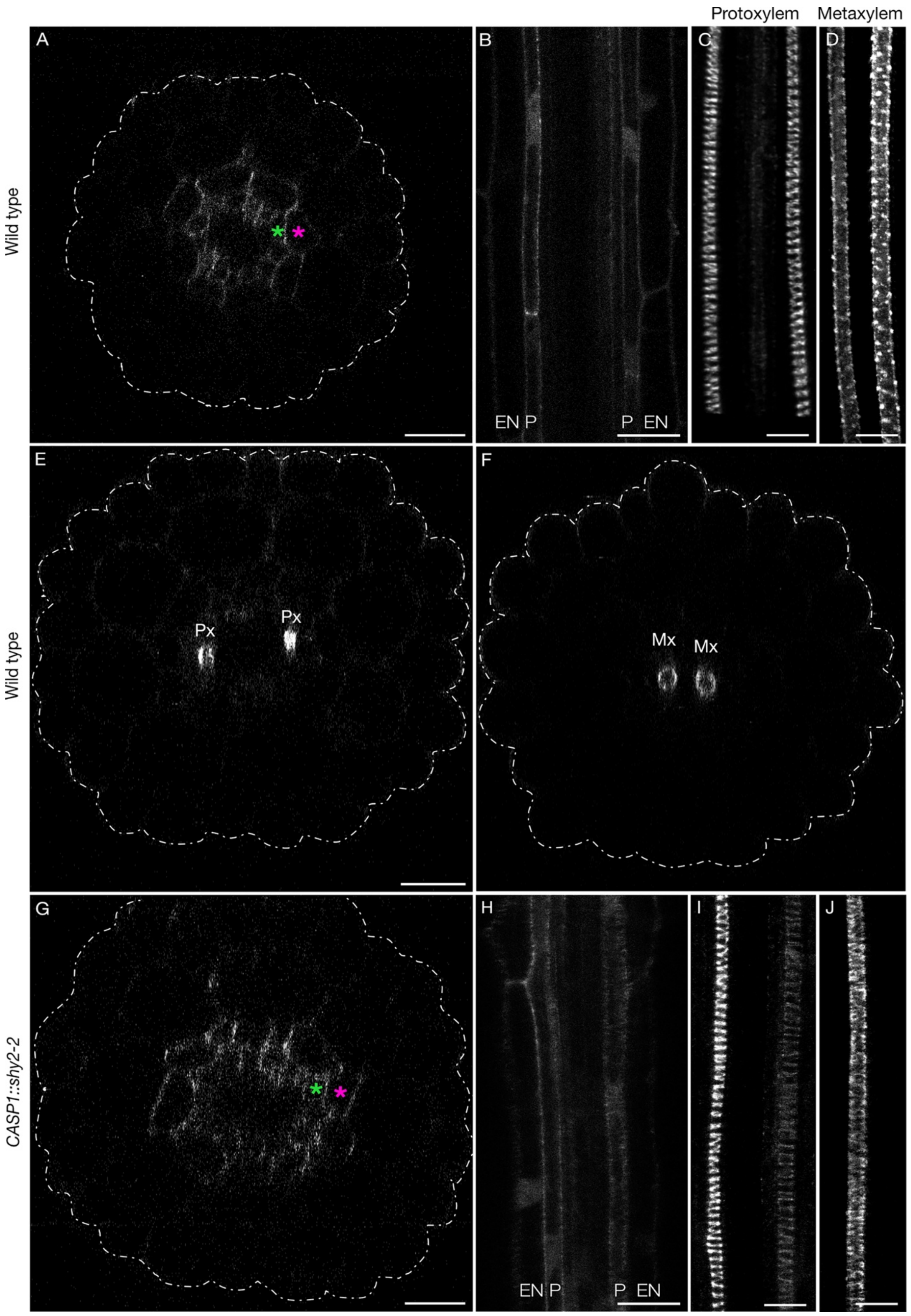
Protoxylem
Metaxylem
A
B
C
D
Wild type
EN P
P EN
E
F
Wild type
Px
Px
Mx
Mx
G
H
I
J
CASP1::shy2-2
EN P
P EN

Fig. S4. ***MAP70-5pro::CITRINE:MAP70-5*** **is expressed in different root cell types.**

Expression of *MAP70-5pro::CITRINE:MAP70-5* in the root of a seven-day-old seedling. (**A, B**) In the root hair development zone, *MAP70-5pro::CITRINE:MAP70-5* is expressed in the pericycle and endodermis. (**C**, **D**) *MAP70-5pro::CITRINE:MAP70-5* is also expressed in differentiating proto- and metaxylem cells, respectively. (**E**, **F**) Cross sections showing CITRINE:MAP70-5 expression in differentiating protoxylem (Px) (**E**) or metaxylem (Mx) (**F**). (**G**, **H**) *MAP70-5pro::CITRINE:MAP70-5* is expressed in the pericycle and endodermis in the root hair development zone in the *CASPpro1::shy2-2* seedlings. (**I**, **J**) *MAP70-5pro::CITRINE:MAP70-5* expression in differentiating protoxylem (**I**) or metaxylem (**J**) cells in *CASP1pro::shy2-2* seedlings. (**A**, **E**-**G**) Orthogonal views of roots in the early differentiation zone. Green asterisks indicate the pericycle and magenta asterisks mark the endodermis. (**A**, **E**-**G**) Cross sections were extracted from confocal z-stacks and dotted white lines indicate the outline of the root. (**C**-**D**, **I-J)** Maximum projection of a z-stack. Scale bars 10 µm (**C**-**D**, **I-J**) and 20 µm (**A**, **B, E**-**H**). P = pericycle, EN = endodermis.

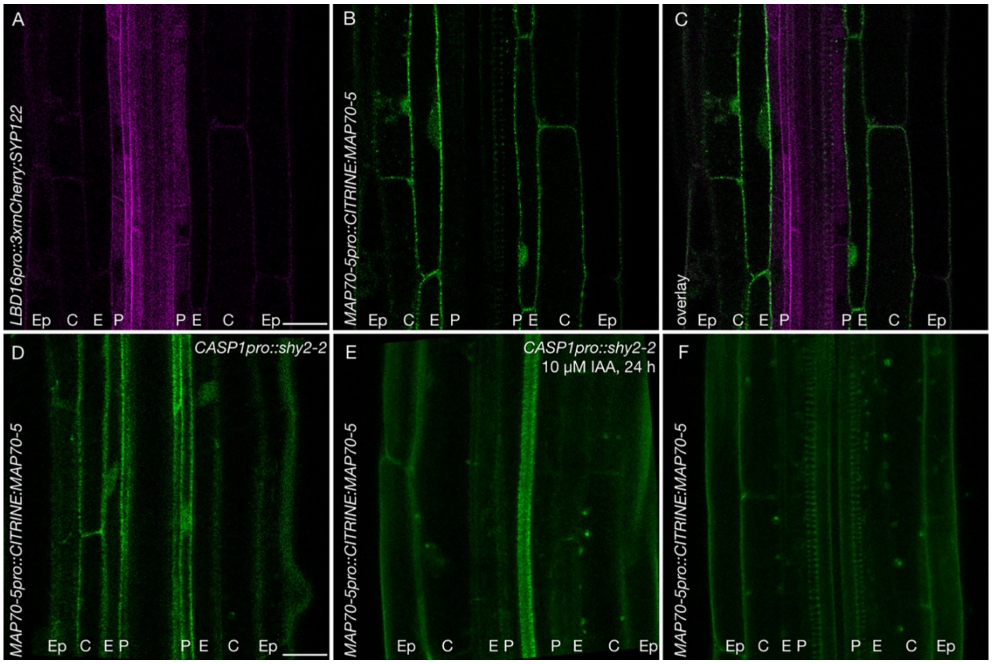

Fig. S5. Endodermal MAP70-5 expression requires Aux/IAA mediated auxin signaling

(**A-C**) Confocal images of seedlings co-expressing the plasma membrane marker *LBD16pro::3xmCherry:SYP122* (**A** and **C**, magenta) and (**B**), *MAP70-5pro::CITRINE:MAP70-5* (**B** and **C**, green) after auxin treatment (10 μM IAA, 24 h) and overlay of both channels (**C**). (**D)** *MAP70-5pro::CITRINE:MAP70-5* expression in *CASP1pro::shy2-2* in the early elongation zone where the *CASP1pro* is not active yet. (**E**) Auxin-mediated induction of *MAP70-5pro::CITRINE:MAP70-5* in the endodermis and the cortex is blocked in *CASP1pro::shy2-2* plants. (**F**) The expression of MAP70-5 in the protoxylem is not affected in *CASP1pro::shy2-2* and IAA treatment (10μM, 24hr). Scale bar is 20 μm. P = pericycle, E = endodermis, C = cortex, Ep = epidermis.

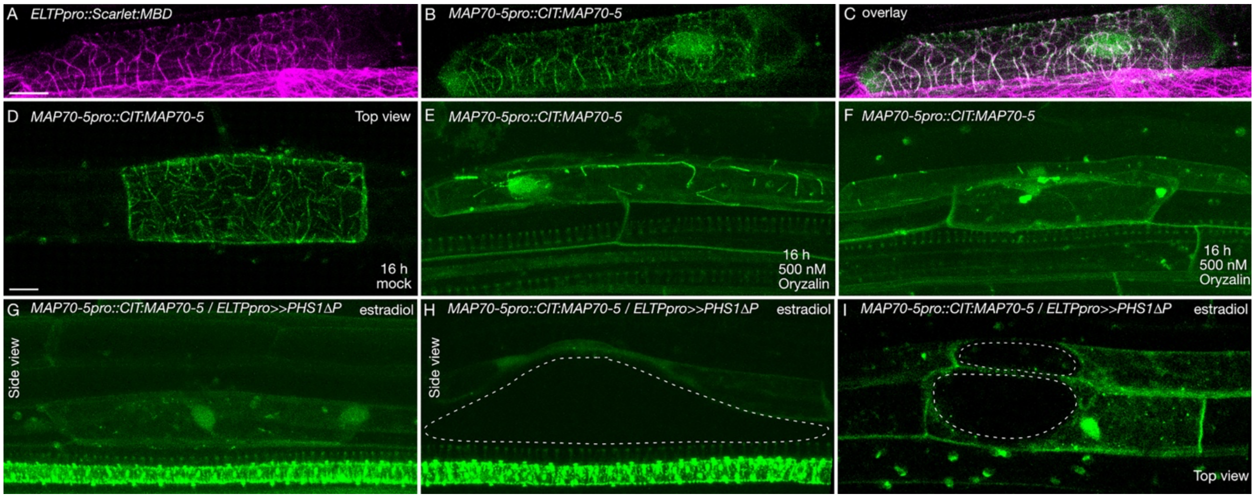

Fig. S6. MAP70-5 partially colocalizes with microtubules and requires an intact CMT cytoskeleton for correct subcellular localization.

(**A, C**) Confocal images of endodermal cells co-expressing *ELTPpro::mScarlet-I:MBD* (grey (**A**), magenta (**C**)) and (**B, C**) *MAP70-5pro::CITRINE:MAP70-5* (grey (**B**), green (**C**)) showing partial colocalization. (**D-F**) CITRINE:MAP70-5 localization after 16h mock (**G**) or oryzalin treatment (500 nM, H and I). (**G**) Stage III/IV, (**H**) stage II/III, and (**I**) stage III/IV LRP. (**J-K**) Disruption of the CMT in the endodermis after *ELTPpro>>PHS1ΔP* induction in *MAP70-5pro::CITRINE:MAP70-5* expressing lines. (**J**) Stage II/III, (**K**) Stage III/IV, and (**L**) Stage IV/V. All images are maximum projections of confocal z-stacks. Scale bar 10 µm.

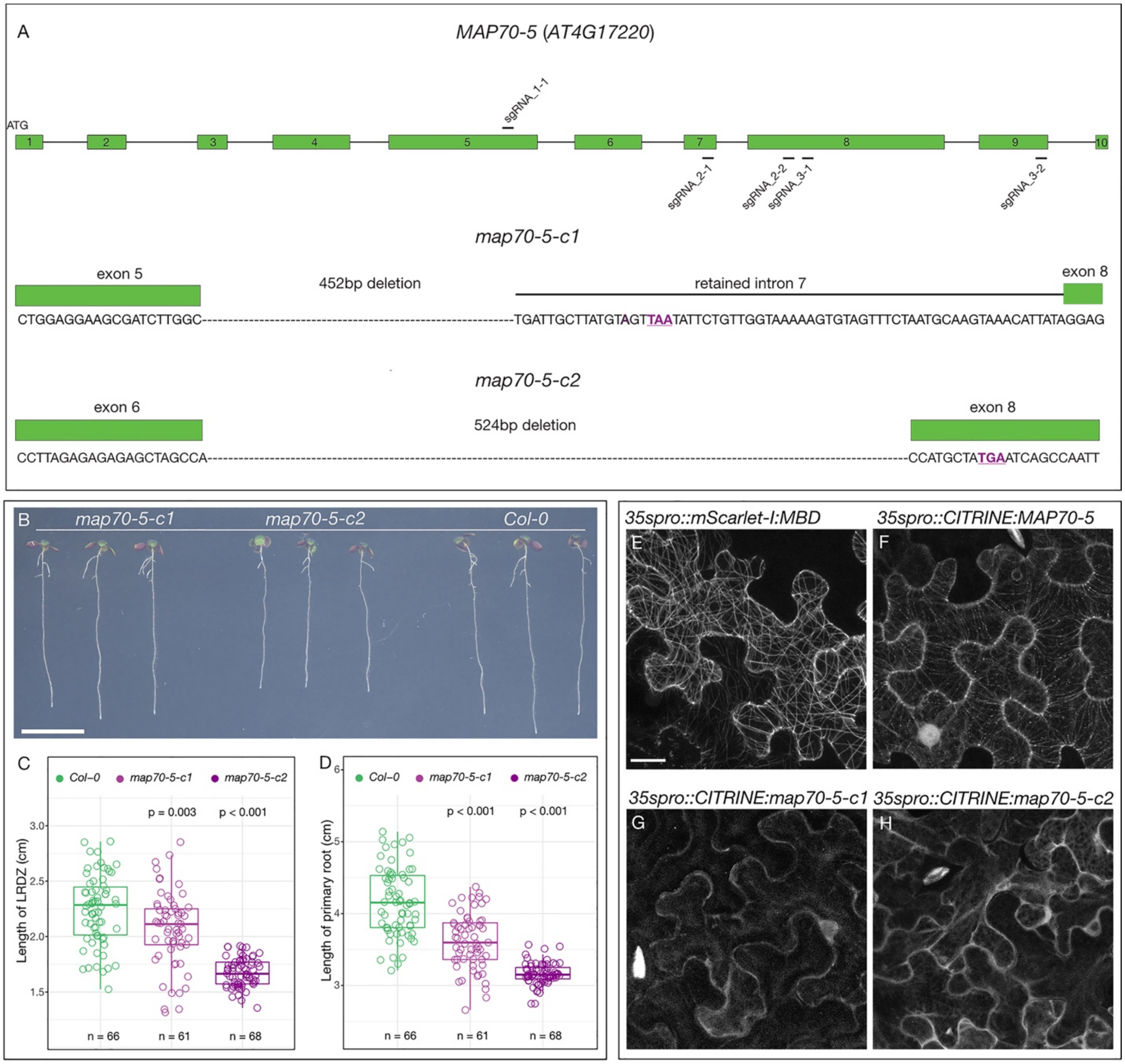

Fig. S7. ***map70-5*** **mutants.**

(**A**) Schematic representation of the gene model of *MAP70-5* and the position of the different sgRNAs used to generate *map70-5* mutants. Bottom panel shows generated deletions that all result in early stop codons, as highlighted in underlined purple letters. (**B**) Image of seven-day old *map70-50-c2, map70-50-c1 and* Col-0 seedlings. Scale bar 1 cm (**C**) Quantification of the LRDZ length and (**D**) of the total root length. Both CRISPR alleles are significantly shorter than the wild type. (**C**, **D**) Two separate student's t-tests on Col-0 and *map70-50-c1 and* Col-0 and *map70-50-c2* were performed. Transient ectopic expression of *35Spro::mScarlet-I:MBD* (**E**), *35Spro:CITRINE:MAP70-5* (**F**) shows labelling of CMTs, whereas transient ectopic expression of

35Spro::CITRINE:map70-5-c1 (**G**) *or* *35Spro::CITRINE:map70-5-c2* (**H**) results in faint cytosolic fluorescence.

Supplemental Table S1. Quantification of LR morphology changes in *map70-5-c1*.

Primordia	Stage I		Stage II		Stage III		Stage IV	
Observations	Wild type	Aberrant	Wild type	Aberrant	Wild type	Aberrant	Wild type	Aberrant
Control (15 roots)	28 (100%)	0 (0%)	26 (76.47%)	8 (23.53%)	8 (75%)	2 (25%)	10 (100%)	0 (0%)
map70-5c1 (28 roots)	56 (85.71%)	9 (14.9%)	17 (23.97%)	57(77.03%)	7 (33.3%)	174 (66,6%)	6 (76.09%)	17 (73.91%)

Observations of mis-shaped primordia and cell division pattern-altered LRPs, and/or delay in endodermis thinning (absolute numbers and percentage). Six to seven day old seedlings were staged until the first primordium pierced the endodermis (mainly stage IV in WT) For visualization of the cell outline, *map70-5-c1* carries the plasma membrane marker *UBQ10pro::EYFP:NPSN12*.

Table S2. Primers used for cloning, CRISPR and genotyping.

Primer name	Sequence
Cloning	
MAP70-5proKpnIfw	CGGGTACCGATCTTCTTCTATATATAC
MAP70-5proKpnIrv	CGGGTACCTTTGTAGTCTCAACGGCTC
attB2r-Map70-5	TGTACAAAGTGGTTATGACTGCAGCAGAAAACC
attB3-Map70-5rv	ATAATAAAGTTGTCTACTGGCAACGTGGATTG
proLBD16-KpnIfw	GGGGTACCTTCCTAAGCCACCTAAGCAG
proLBD16-KpnIrv	GGGGTACCCGGCGAAACGAACAAAAAAG
CRISPR	
sg70-5_1-fw	attgGAGGAAGCGATCTTGGCAGG
sg70-5_1-rv	aaacCCTGCCAAGATCGCTTCCTC
sg70-5_2-1fw	gtcaTAGCTGGGACTCAGCCTTAG
sg70-5_2-1rv	aaacCTAAGGCTGAGTCCCAGCTA
sg70-5_2-2fw	gtcaTTAGGATTCTTGACCAGTGG
sg70-5_2-2rv	aaacCCACTGGTCAAGAATCCTAA
sg70-5_3-1fw	attgCAGCTAAGAGGCTCAGTTAC
sg70-5_3-1rv	aaacGTAACTGAGCCTCTTAGCTG
sg70-5_3-2fw	attgGAAAGGCAAAACTACCGAGA
sg70-5_3-2rv	aaacTCTCGGTAGTTTTGCCTTTC
Genotyping	
oJV516	GAAAATTAACCGCCAGAAAGTG
oJV573	GGCTTTGCAATCCGATATAC

Movie captions.

Supplemental Movie S1. CITRINE:MAP70-5 dynamics during metaxylem differentiation.

Confocal time-lapse of a five day old seedling showing CITRINE:MAP70-5 fluorescence in the metaxylem and in the endodermal cell overlying a stage III primordium. Please not the disappearance of the CITRINE signal when metaxylem cells undergo programmed cell death. In contrast, the CITRINE fluorescence in the endodermal cell is not affected. Images are maximum projections of z-stacks that were acquired in 30 minutes intervals over 16 h. Scale bar is 20 µm.

Supplemental Movie S2. MAP70-5 dynamics in endodermal cells overlying stage II LRP.

Confocal time-lapse of a five-day old seedling showing CITRINE:MAP70-5 dynamics in the endodermal cell overlying a stage II primordium. Images are maximum projections of z-stacks that were acquired in 15 minutes intervals over 14.5 h. Scale bar is 20 µm

Supplemental Movie S3. MAP70-5 localization in endodermal cells overlying stage IV/V LRP

Confocal time-lapse of a five-day old seedling showing CITRINE:MAP70-5 fluorescence in the endodermal cell overlying a stage II primordium. Images are maximum projections of z-stacks that were acquired in 30 minutes intervals over 6.5 h. Scale bar is 20 µm
